# Supplementary material for: Combined impact of pesticides and other environmental stressors on animal diversity in irrigation ponds
Source: PLoS One. 2020 Jul 2;15(7):e0229052. doi: 10.1371/journal.pone.0229052 (PMC7332035; doi:10.1371/journal.pone.0229052)
Supplement: S1 Appendix — (PDF) [file pone.0229052.s002.pdf]

Hiroshi C. Ito, Hiroaki Shiraishi, Megumi Nakagawa, and Noriko Takamura. Combined impact of pesticides and other environmental stressors on animal diversity in irrigation ponds.

S1 Appendix: Measurement of physicochemical properties

S1.1 Pesticides

Among the 31 pesticides analyzed in this study, which were selected by considering spray timing and type of pesticide, 28 were analyzed by high-performance liquid chromatography–tandem mass spectrometry (HPLC-MS/MS) and 3 were detected with gas chromatography–mass spectrometry (GC-MS) (Table S2-1 in supplemental material).

Water samples were directly collected at 20-cm depth in 1-gallon umber-colored glass bottles that had been rinsed with the same water from the reservoirs. The collected samples were transported to the laboratory under cool dark conditions and then filtered through a glass fiber filter (GF/F, Whatman). The filtrate was refrigerated at 4 °C and extracted as soon as possible.

HPLC-MS/MS

Pesticides were extracted by passing 200 mL of water through a solid-phase extraction column (BondElutC18, Varian) preconditioned with 8 mL of acetone and 5 mL of water. The column was washed with 5 mL of pure water (Milli-Q), and then the pesticide was eluted with 8 mL of acetone. The extract was concentrated under a gentle nitrogen stream to just dry, then dissolved in 500 μL methanol, to which we added 500 μL of pure water and mixed well. Pesticides were analyzed by HPLC-MS/MS operated in the electrospray ionization and selected reaction monitoring (SRM) mode, and 5 μL of the solution was injected into the HPLC-MS/MS. The calibration curve was constructed with the peak area of SRM ions. The recovery rate of each pesticide exceeded 80%, and the quantitative value was not corrected by the recovery rate. Only E-isomer of pyriminobac-methyl was quantified.

The mass spectrometer used was a Quattro Ultima (Micromass), HPLC was an Agilent 1100

(Agilent Technologies), the column was an Atlantis T3 (3 μm, 2.1 mm i.d. × 150 mm, Waters), mobile phase A was 5 mM ammonium acetate/water, and mobile phase B was 5 mM ammonium acetate/methanol. Mobile phase B was increased from 15% to 95% over 17.5 min and then held for 12.5 min. The flow rate was 0.2 mL min^{-1}.

Conditions of LC-MS/MS are shown in Table S2-2 in the supplemental material (Pizzutti et al. 2007).

GC-MS

Pesticides were extracted by passing 500 mL of water through a solid-phase extraction column (BondElutC18, Varian) preconditioned with 8 mL of acetone and 5 mL of water. The column was washed with 5 mL of pure water and then the pesticide was eluted with 8 mL of acetone. The extract was dried under a nitrogen stream, dissolved in 250 μL of acetone, and then mixed well with 250 μL hexane solution of internal standard (4-chlorotoluene-d_4, 1,4-dichlorobenzene-d_4, naphthalene-d_8, phenanthrene-d_{10}, fluoranthene-d_{10}, acenaphthene-d_{10}, chrysene-d_{12}, pellerin-d_{12}, 1 ppm). We injected 1 μL of the solution into the GC-MS/MS, operated in the electron ionization and SRM mode. The concentration of each pesticide was calculated by the internal standard method. The recovery of each pesticide exceeded 80%. The quantitative value was not corrected by the recovery rate.

The mass spectrometer used was an Agilent 5973N (Agilent Technologies), and GC was an Agilent 6890 (Agilent Technologies) equipped with a splitless injector. The ion source temperature was 230 °C, the inlet temperature was 250 °C, and the interface temperature was 280 °C. A fused silica capillary column DB-5 MS (30 m × 0.25 mm i.d., film thickness 0.25 μm, Agilent) was used for separation. After injection, the initial column oven temperature of 80 °C was kept for 1 min, then raised at a rate of 10 ° C/min to 280 °C, and held for 10 min. Helium was used as a carrier gas at the flow rate of 1 mL min^{-1}.

Table S2-3 in the supplemental material gives the conditions of GS-MS (Uno et al. 2001).

References

Pizzutti, I. R., A. de Kok, R. Zanella, M. B. Adaime, M. Hiemstra, C. Wickert, and O. D. Prestes. 2007. Method validation for the analysis of 169 pesticides in soya grain, without cleanup, by liquid chromatography-tandem mass spectrometry using positive and negative electrospray ionization. Journal of Chromatography A 1142:123–136.

Uno, S., H. Shiraishi, S. Hatakeyama, A. Otsuki, and J. Koyama. 2001. Accumulative characteristics of pesticide residues in organs of bivalves (*Anodonta woodiana* and *Corbicula leana*) under natural conditions. Archives of Environmental Contamination and Toxicology 40(1):35–47.

S1.2 Physicochemical properties

Monitoring sites were at the inlet of a waterway entering the irrigation pond. The water temperature and pH were measured just above the bottom sediment using a multi-water quality monitoring system (U-21 DX, Horiba). Pond water at 20-cm depth was directly sampled into 2-L polypropylene bottles and 100-mL vials, and transported to the laboratory under cool dark conditions.

Analysis of water quality

The concentration of chlorophyll *a* (Chl-a) was determined from the absorbance of the methanol extract of suspended solids (SS) at 665 nm, measured with a spectrophotometer (U-2800A, Hitachi) (Marker et al. 1980). The SS concentration was obtained by weighing SS on a filter (GF/F, Whatman).

Ammonia nitrogen (NH_4-N) was determined by the indophenol method. Nitrite nitrogen (NO_2-N) and nitrate nitrogen (NO_3-N) were determined by the naphthylethylene diamine method (APHA 1998) using an AutoAnalyzer (AACS II, Bran + Luebbe), where NO_3^- is

reduced on a cadmium column to NO_2^-. The total nitrogen (TN) was measured after alkaline potassium peroxodisulfate oxidation and conversion of resulting NO_3^- to NO_2^-. The soluble reactive phosphorus concentration was measured according to the molybdenum blue method using an AutoAnalyzer (APHA 1998). Total phosphorus (TP) was determined as soluble reactive phosphorus after acidic peroxodisulfate oxidation.

For measuring ignition loss, the mud was freeze-dried and then mixed well by using a medicine spoon. Five grams of the mud was then put in the oven for the measurement of weight loss on ignition at 550 °C for 1 h. After cooling to room temperature, the sample was weighed again to calculate the loss.

On 13–15 May 2007, pond depth was measured with a ruler with a spindle at the deepest point of each pond. Pond area and concrete bank rate were measured from geo-referenced 1:2500 geographic maps on ArcGIS 9.1. Percent coverage of floating-leaved plants and of emergent plants in each pond surface area was measured on aerial photographs taken in August 2006. Intensities of pond drainage were classified into three categories (0: no drainage, 1: partial drainage, 2: full drainage) based on interviews with pond owners. Densities of invasive alien species were classified into two categories (0: low, 1: high) based on whether the sampling of freshwater animals found those species or not.

References

APHA. 1998. Standard Methods for the Examination of Water and Wastewater, 20th edn. American Public Health Association, Washington.

Marker, A. F. H., E. A. Nusch, H. Rai, and B. Riemann. 1980. The measurement of Photosynthetic pigments in freshwaters and standardization of methods: conclusions and recommendations. Advances in Limnology,, 14: 91–106.
