# Supplementary material for: Combined impact of pesticides and other environmental stressors on animal diversity in irrigation ponds
Source: PLoS One. 2020 Jul 2;15(7):e0229052. doi: 10.1371/journal.pone.0229052 (PMC7332035; doi:10.1371/journal.pone.0229052)
Supplement: S2 Appendix — (PDF) [file pone.0229052.s003.pdf]

Hiroshi C. Ito, Hiroaki Shiraishi, Megumi Nakagawa, and Noriko Takamura. Combined impact of pesticides and other environmental stressors on animal diversityin irrigation ponds.

S2 Appendix: Statistical analysis

S2.1 Principal components for the large contraction group

In our statistical analysis, the large contraction group, denoted here by “Cont”, was represented by its four principal components, denoted by “Cont.var1”, “Cont.var2”, “Cont.var3”, and “Cont.var4”, as grouped variables. For each grouped variable, we list here the 10 uncontracted environmental variables with the highest absolute correlations with it (“I.”, “H.”, and “F.” mean insecticide, herbicide, and fungicide, respectively).

Cont.var1: I.thiamethoxam (–0.86), H.bromobutide (–0.83), H.bentazone (–0.83), TP (–0.81), F.pyroquilon (–0.77), H.oxaziclomefon (–0.75), F.tiadinil (–0.72), F.furametpyr (–0.69), H.chlomeprop (–0.68), TN (–0.67);

Cont.var2: E-plant noncoverage (0.80), I.malathion (–0.77), I.tebufenozide (–0.75), I.buprofezin (–0.64), SS (–0.61), H.pentoxazone (0.55), H.butachlor (0.51), F.isoprothiolane (0.50), I.imidacloprid (0.48), F.pyroquilon (–0.48);

Cont.var3: H.butachlor (–0.70), F.fthalide (–0.61), black bass (–0.59), F.ferimzone (–0.59), WT (0.58), F.isoprothiolane (–0.50), I.tebufenozide (–0.49), H.pentoxazone (–0.49), I.clothanidin (–0.47), I.malathion (–0.47);

Cont.var4: Chl-a (0.69), pH (0.68), H.oxaziclomefon (–0.43), H.chlomeprop (–0.42), F.azoxystrobin (–0.41), SS (0.40), TN (0.39), H.pyriminobac_methyl_E (0.39), F.furametpyr (0.37), H.mefenacet (0.37).

S2.2 Model selection

We used the 14 contracted environmental variables as the explanatory variables to explain the response variable, taxonomic richness of a focal animal category. For convenience, all explanatory variables were rescaled to range from 0 to 1 (their mean and standard deviation

could deviate from 0 and 1 after this operation). For each of the possible subsets of the 14 explanatory variables, we constructed a Poisson regression mixed model (Broström and Holmberg 2011), where any model has at least one explanatory variable. In each model, the response variables were described by a vector $\mathbf{y} = (y_1, \dots, y_M)$ of length $M = 21$ (the number of studied ponds), where y_i is its value for the ith pond. Explanatory variables were described by a set of vectors $\mathbf{x}_1, \dots, \mathbf{x}_K$ with $1 \leq K \leq 14$, each of which was denoted by $\mathbf{x}_k = (x_{k,1}, \dots, x_{k,M})$. We assumed that y_i follows the Poisson distribution,

$$y_i \quad \sim \quad \mathrm{Poisson}(Y_i) \qquad \text{Eq. (1) in the main text}$$

with its mean Y_i described as

$$\ln(Y_i) \quad = \quad \alpha + \sum_{k=1}^{K} \beta_k x_{k,i} + r_i, \qquad \text{Eq. (2) in the main text}$$

where α is the intercept, $x_{k,i}$ is the intensity of the kth explanatory variable at the ith pond with its regression coefficient β_k, and r_i is a pond-specific random effect. r_i follows the normal distribution with average 0 and standard deviation σ. For each of the models constructed above, we calculated maximum likelihood estimations for α, $\beta_1, \dots, \beta_K$, maximum marginal-likelihood estimation for σ (Broström and Holmberg 2011), and the Akaike information criterion (AIC) (Akaike 1973). To suppress the estimation bias of AIC as a distance measure from an unknown true model, we excluded models that had more free parameters than one-third of the sample size (Kitagawa et al. 1983); models with $M/3 < K + 2$ (i.e., $\beta_1, \dots, \beta_K$, α, and σ) were excluded. We also fitted the normal Poisson regression model by setting $\sigma = 0$ in advance, in which case models with $M/3 < K + 1$ (i.e., $\beta_1, \dots, \beta_K$, α) were excluded.

When the model with the lowest AIC, referred to as the contracted best model, had residuals with significant spatial autocorrelation (i.e., p-value < 0.05 in either Moran's I test or Geary's C test), we excluded the model because the assumption of independence was violated, and we treated the second best model as the contracted best model. This operation was repeated until the spatial autocorrelation in the contracted best model's residuals became non-significant. (For the results reported in this paper, none of the initial best models had residuals with significant

spatial autocorrelation.)

S2.3 Statistical significance for statistically contributive stressors

In this study, statistical significance for a statistically contributive stressor was evaluated by a permutation test that explicitly repeats the model selection, as explained below.

Test statistic and p-*value*

We denote the observed values for the response and explanatory variables (contracted environmental variables) by $\mathbf{y}$ and $\mathbf{x}_1, \ldots, \mathbf{x}_H$ with $H = 14$, respectively. We describe the best model for the observed data $\mathbf{y}, \mathbf{x}_1, \ldots, \mathbf{x}_H$ by $\mathbf{m} = (m_1, \ldots, m_H)$ with 0 or 1 for each entry, where $m_h = 1$ means inclusion of the hth explanatory variable in the best model. We assume without loss of generality that the Hth explanatory variable is to be tested, by its random resampling for J times. In the jth resampling of $\mathbf{x}_H$ for $j = 1, \ldots, J$, we denote the resampled values by $\tilde{\mathbf{x}}_H^j$, and denote the best model for $\mathbf{y}, \mathbf{x}_1, \ldots, \tilde{\mathbf{x}}_H^j$ by $\tilde{\mathbf{m}}^j = (\tilde{m}_1^j, \ldots, \tilde{m}_H^j)$. We use a test statistic described as

$$A(\tilde{\mathbf{x}}_H^j) = \begin{cases} \delta\mathrm{AIC}(\tilde{\mathbf{x}}_H^j; \tilde{\mathbf{m}}^j) & \text{for} \quad \text{condition (a) satisfied} \\ -\infty & \text{for} \quad \text{otherwise} \end{cases}, \qquad \text{(S2.1)}$$

with condition (a): conditions (i) and (iii) for the statistical contributiveness (defined in "Statistical inference" in the main text) are both satisfied, and the best model $\tilde{\mathbf{m}}^j$ for $\mathbf{y}, \mathbf{x}_1, \ldots, \tilde{\mathbf{x}}_H^j$ includes the Hth explanatory variable ($\tilde{m}_H^j = 1$) with a negative regression coefficient.

Here, $\delta\mathrm{AIC}(\tilde{\mathbf{x}}_H^j; \tilde{\mathbf{m}}^j)$ is the AIC difference caused by dropping the Hth explanatory variable from $\tilde{\mathbf{m}}^j$, given by

$$\delta\mathrm{AIC}(\tilde{\mathbf{x}}_H^j; \tilde{\mathbf{m}}^j) = \mathrm{AIC}(\mathbf{y}, \mathbf{x}_1, \ldots, \mathbf{x}_{H-1}, \tilde{\mathbf{x}}_H^j; \tilde{\mathbf{m}}^{j,H-}) - \mathrm{AIC}(\mathbf{y}, \mathbf{x}_1, \ldots, \mathbf{x}_{H-1}, \tilde{\mathbf{x}}_H^j; \tilde{\mathbf{m}}^j), \qquad \text{(S2.2)}$$

with $\tilde{\mathbf{m}}^{j,H-} = (\tilde{m}_1^j, \ldots, \tilde{m}_{H-1}^j, 0)$.

Since the Hth explanatory variable is a statistically contributive stressor, satisfying condition (a) for $\mathbf{y}, \mathbf{x}_1, \ldots, \mathbf{x}_H$, and $\mathbf{m}$, we see

$$\begin{aligned} A(\mathbf{x}_H) &= \delta\mathrm{AIC}(\mathbf{x}_H; \mathbf{m}) \\ &= \mathrm{AIC}(\mathbf{y}, \mathbf{x}_1, \dots, \mathbf{x}_{H-1}, \mathbf{x}_H; \mathbf{m}^{H-}) - \mathrm{AIC}(\mathbf{y}, \mathbf{x}_1, \dots, \mathbf{x}_{H-1}, \mathbf{x}_H; \mathbf{m}) \end{aligned} \quad \text{(S2.3)}$$

with $\mathbf{m}^{H-} = (m_1, \dots, m_{H-1}, 0)$. Then by counting $A(\tilde{\mathbf{x}}_H^j)$ that are no less than $A(\mathbf{x}_H)$, we calculate a *p*-value as

$$p(A(\mathbf{x}_H)) = \frac{1}{J}\sum_{j=1}^{J} \mathrm{count}^+\left(A(\tilde{\mathbf{x}}_H^j) - A(\mathbf{x}_H)\right), \quad \text{(S2.4)}$$

with

$$\mathrm{count}^+(t) = \begin{cases} 1 & \text{for} \quad t > 0 \\ 0.5 & \text{for} \quad t = 0 \\ 0 & \text{for} \quad t < 0 \end{cases}. \quad \text{(S2.5)}$$

Note that this permutation test corresponds to a one-sided test because condition (a) requires a negative regression coefficient for the focal explanatory variable. Allowing both signs gives a two-sided test.

To see the connection with normal permutation tests for regression coefficients in a given model, we assume that $\tilde{\mathbf{m}}^j$ is always equal to $\mathbf{m}$ for all $j = 1, \dots, J$, and we neglect condition (a). Then we can transform $A(\tilde{\mathbf{x}}_H^j)$ as

$$\begin{aligned} A(\tilde{\mathbf{x}}_H^j) &= \delta\mathrm{AIC}(\tilde{\mathbf{x}}_H^j; \mathbf{m}) \\ &= \mathrm{AIC}(\mathbf{y}, \mathbf{x}_1, \dots, \mathbf{x}_{H-1}, \tilde{\mathbf{x}}_H^j; \mathbf{m}^{H-}) - \mathrm{AIC}(\mathbf{y}, \mathbf{x}_1, \dots, \mathbf{x}_{H-1}, \tilde{\mathbf{x}}_H^j; \mathbf{m}) \\ &= -2l\left((\mathbf{y}, \mathbf{x}_1, \dots, \mathbf{x}_{H-1}, \tilde{\mathbf{x}}_H^j; \mathbf{m}^{H-})\right) + 2l(\mathbf{y}, \mathbf{x}_1, \dots, \mathbf{x}_{H-1}, \tilde{\mathbf{x}}_H^j; \mathbf{m}) - 2, \end{aligned} \quad \text{(S2.6)}$$

where $l(\mathbf{y}, \mathbf{x}_1, \dots, \mathbf{x}_{H-1}, \tilde{\mathbf{x}}_H^j; \mathbf{m})$ is the maximum log-likelihood for model $\mathbf{m}$ and dataset $\mathbf{y}, \mathbf{x}_1, \dots, \mathbf{x}_{H-1}, \tilde{\mathbf{x}}_H^j$. We see $A(\mathbf{x}_H) = -2l(\mathbf{y}, \mathbf{x}_1, \dots, \mathbf{x}_{H-1}, \tilde{\mathbf{x}}_H^j; \mathbf{m}^{H-}) +$ $2l(\mathbf{y}, \mathbf{x}_1, \dots, \mathbf{x}_{H-1}, \mathbf{x}_H; \mathbf{m}) - 2$. Thus, in this case, $p(A(\mathbf{x}_H))$ gives a *p*-value by a permutation test using the log-likelihood ratio for the test statistic in a given model $\mathbf{m}$, without taking into account the model selection process.

Resampling rule

In our permutation test, following the philosophy of the permutation-of-regressor-residuals test (Werft and Benner 2010), we keep the expected correlation structure among all explanatory

variables constant in the contracted best model $\mathbf{m} = (m_1, \ldots, m_H)$. We express $\mathbf{x}_H$ as a linear function of the remaining explanatory variables and residuals $\boldsymbol{\varepsilon}_H$,

$$\mathbf{x}_H \;=\; m_0\eta_0 + m_1\eta_1\mathbf{x}_1 + \ldots + m_{H-1}\eta_{H-1}\mathbf{x}_{H-1} + \boldsymbol{\varepsilon}_H, \qquad \text{(S2.7)},$$

where $m_0 = 1$. For $h = 1, \ldots, H$, only η_h with $m_h = 1$ are specified by the least squares estimations ($\mathbf{x}_h$ with $m_h = 0$ are not used for the regression). Then random permutation of the residuals $\boldsymbol{\varepsilon}_H$ by J times gives

$$\tilde{\mathbf{x}}_H^j \;=\; m_0\eta_0 + m_1\eta_1\mathbf{x}_1 + \ldots + m_{H-1}\eta_{H-1}\mathbf{x}_{H-1} + \tilde{\boldsymbol{\varepsilon}}_H^j \qquad \text{(S2.8)}$$

for $j = 1, \ldots, J$, with the jth permutated residual $\tilde{\boldsymbol{\varepsilon}}_H^j$.

S2.4 Discussion on conditions for statistical contributiveness

In condition (i) for the statistical contributiveness (defined in "Statistical inference" in the main text), the threshold $\mathrm{C}_{\Delta\mathrm{AIC}} = 2.0$ is chosen because any model with $\Delta\mathrm{AIC} > 2.0$ is rejected by the parametric likelihood ratio test for significance level 0.05, when that model is nested in the contracted best model, as noted by Akaike (1974). This is because the *p*-value in such a case is given by $\Pr\,(\chi_k^2 > \Delta\mathrm{AIC} + 2k)$ in the parametric log-likelihood test, where and χ_k^2 is a chi-square random variable with k degrees of freedom, i.e., difference in the number of explanatory variables between the contracted best model and a focal nested model (Murtaugh 2014). $\Pr(\chi_k^2 > \Delta\mathrm{AIC} + 2k) < 0.05$ holds good for any $k \geq 1$ under $\Delta\mathrm{AIC} > 2.0$. Although this relationship does not hold for non-nested models, choosing 2.0 seems to be a good starting point. Condition (ii) reinforces condition (i) by suppressing biases caused by small sample sizes, because the permutation-of-regressor-residuals test is reportedly robust against small sample sizes and correlations among explanatory variables (Potter 2005, Werft and Benner 2010). (When an $\alpha_{\Delta\mathrm{AIC}}$ lower than 0.05 is chosen, $\mathrm{C}_{\Delta\mathrm{AIC}}$ needs adjustment so that any nested model of $\Delta\mathrm{AIC} > \mathrm{C}_{\Delta\mathrm{AIC}}$ is rejected by the parametric likelihood ratio test for the significance level $\alpha_{\Delta\mathrm{AIC}}$.) As for condition (iii), its examination is straightforward if the contracted best model in (i) has no grouped variable, otherwise it becomes somewhat complicated (see the next

subsection for details).

Condition (i) examines whether all models with $\Delta\text{AIC} \leq 2.0$ have the focal explanatory variable with the same sign for its regression coefficients. If models with $\Delta\text{AIC} \leq 2.0$ include all models with $p \geq 0.05$ (i.e., models not rejected in comparison with the best model), or equivalently if $\Delta\text{AIC} > 2.0$ ensures $p < 0.05$, then our definition for the statistical contributiveness has a straightforward connection with the standard concept of statistical significance. This can be stated as "the focal explanatory variable has the same sign of effect in all models that may not be rejected by statistical tests in comparison with the best model." However, $\Delta\text{AIC} > 2.0$ ensures $p < 0.05$ only for models nested in the best model, in the parametric likelihood-ratio test, as explained above. We can calculate p-values for non-nested models by applying the Cox test (Cox 1961), Vuong test (Vuong 1989), or Clarke test (Clarke 2003). In this case, however, the number of models with $p \geq 0.05$ can be much larger than that of models with $\Delta\text{AIC} \leq 2.0$, which may result in too conservatively judging the statistical contributiveness of explanatory variables. Using permutation tests instead might give tighter p-values, but it slows the analysis considerably. Consequently, for quick extraction of meaningful information from the data, we adopted $\Delta\text{AIC} \leq 2.0$ instead of $p > 0.05$ in condition (i).

S2.5 Condition (iii) for statistical contributiveness

We explain how to examine condition (iii) when the contracted best model has grouped variables. We denote the explanatory variables for the contracted best model by $\mathbf{x}_1, \dots, \mathbf{x}_K$, and denote the uncontracted environmental variables by $\mathbf{z}_1, \dots, \mathbf{z}_L$. For $k = 1, \dots, K$, when $\mathbf{x}_k$ is a single variable, then among $\mathbf{z}_1, \dots, \mathbf{z}_L$ we can find $\mathbf{z}_{c(k)}$ that is identical to $\mathbf{x}_k$, and we include it in the initial model for the stepwise selection by AIC. When $\mathbf{x}_k$ is a grouped variable representing a contraction group having more than two uncontracted environmental variables, we choose $\mathbf{z}_{c(k)}$ so that its absolute correlation with $\mathbf{x}_k$ is the maximum among $\mathbf{z}_1, \dots, \mathbf{z}_L$, that is, $c(k) = \text{argmax}_{j \in \{1,\dots,L\}}(|\text{cor}(\mathbf{x}_k, \mathbf{z}_j)|)$, and we include $\mathbf{z}_{c(k)}$ in the place of $\mathbf{x}_k$ in the

initial model. When $\mathbf{x}_k$ is a grouped variable representing a contraction group having exactly two uncontracted environmental variables, denoted by $\mathbf{z}_{c1(k)}$ and $\mathbf{z}_{c2(k)}$, then both variables always have the same absolute correlation with $\mathbf{x}_k$. Thus, we include both variables for the initial model in the place of $\mathbf{x}_k$.

After obtaining the uncontracted best model by the stepwise model selection, we examine condition (iii) for each of $\mathbf{x}_k$ for $k = 1, \ldots, K$, as follows. When $\mathbf{x}_k$ is a single variable, then we judge that condition (iii) is met if $\mathbf{z}_{c(k)} = \mathbf{x}_k$ is included in the uncontracted best model, and if the regression coefficient $\gamma_{c(k)}$ for $\mathbf{z}_{c(k)}$ in the uncontracted best model shares the same sign with the regression coefficient β_k for $\mathbf{x}_k$ in the contracted best model, that is, $\gamma_{c(k)}\beta_k > 0$.

When $\mathbf{x}_k$ is a grouped variable representing a contraction group having more than two uncontracted environmental variables, then we judge that condition (iii) is met if $\mathbf{z}_{c(k)}$ is included in the uncontracted best model, and if its regression coefficient $\gamma_{c(k)}$ multiplied by $\mathrm{cor}(\mathbf{z}_{c(k)}, \mathbf{x}_k)$ shares the same sign with the regression coefficient β_k for $\mathbf{x}_k$ in the contracted best model, that is, $\gamma_{c(k)}\mathrm{cor}(\mathbf{z}_{c(k)}, \mathbf{x}_k)\beta_k > 0$.

When $\mathbf{x}_k$ is a grouped variable representing a contraction group having exactly two uncontracted environmental variables $\mathbf{z}_{c1(k)}$ and $\mathbf{z}_{c2(k)}$, then we judge that condition (iii) is met if (a) $\mathbf{z}_{c1(k)}$ and $\mathbf{z}_{c2(k)}$ are both included and $\gamma_{c1(k)}\mathrm{cor}(\mathbf{z}_{c1(k)}, \mathbf{x}_k)\beta_k > 0$ and $\gamma_{c2(k)}\mathrm{cor}(\mathbf{z}_{c2(k)}, \mathbf{x}_k)\beta_k > 0$ are both satisfied, or if (b) only $\mathbf{z}_{c1(k)}$ is included and $\gamma_{c1(k)}\mathrm{cor}(\mathbf{z}_{c1(k)}, \mathbf{x}_k)\beta_k > 0$ is satisfied, or if (c) only $\mathbf{z}_{c2(k)}$ is included and $\gamma_{c2(k)}\mathrm{cor}(\mathbf{z}_{c2(k)}, \mathbf{x}_k)\beta_k > 0$ is satisfied.

S2.6 Interaction among statistically contributive explanatory variables

When a focal animal category had more than one statistically contributive explanatory variable in the analysis for main effects (described in “Statistical inference” in the main text), we further

analyzed interactions among them. First, for each possible combination of the contributive variables, we calculated the product of the two variables' intensities at each pond and added it to the set of contracted environmental variables and to the set of uncontracted environmental variables. Second, we conducted the analysis described in the sections "Model selection" and "Statistical inference" in the main text. Note that the set of models examined in this analysis for interactions includes the set of models in the analysis for main effects. Thus, AICs of the contracted best models in this analysis for interactions are always no higher than those of the corresponding contracted best models in the analysis for main effects. Therefore, the contracted best models with interactions are all as good as the corresponding contracted best models without interactions.

S2.7 Best models

The best models for explaining the taxonomic richness of animal groups shown in Figs 2 and 3 in the main text are listed below. All of the best models were not Poisson regression mixed models but normal Poisson regression models (i.e., $\sigma = 0$, which gives $r_i = 0$ for all *i* in Eq. (2) in the main text). Each n_{model} indicates the number of models satisfying $\Delta\mathrm{AIC} \leq 2.0$. "XXX" between two variable names means the interaction of those variables. Statistically contributive stressors in contracted best models are indicated with "*" (contributive and significant) or "%" (contributive but non-significant).

All-sampled

Contracted best model ($r^2 = 0.64,\ n_{\mathrm{model}} = 6$):

E(ln(*y*)) = 4.71 – 0.32[Cont.var1.1] – 0.27[Cont.var1.3] – 0.49[I.BPMC] – 0.61[%shallowness] – 0.54[*F-plant noncoverage] – 0.38[concrete bank]

Uncontracted best model ($r^2 = 0.66$):

E(ln(*y*)) = 4.15 + 0.31[I.thiamethoxam] + 0.32[H.butachlor] – 0.44[I.BPMC] –

0.56[shallowness] – 0.47[F-plant noncoverage] – 0.40[concrete bank]

Large animal

Contracted best model ($r^2 = 0.69,\ n_{\mathrm{model}} = 32$)

E(ln(*y*)) = 4.07 – 0.68[Cont.var1.2] + 0.70[F.IBP...ignition loss] – 1.28[*shallowness] – 0.67[*F-plant noncoverage]

Uncontracted best model ($r^2 = 0.83$)

E(ln(*y*)) = 4.27 – 0.70[E-plant noncoverage] + 0.74[ignition loss] – 1.44[shallowness] – 0.62[F-plant noncoverage] + 0.89[F.furametpyr] – 0.38[F.metominostrobin Z]

Small animal

Contracted best model ($r^2 = 0.59,\ n_{\mathrm{model}} = 18$)

E(ln(*y*)) = 3.71 – 0.37[Cont.var1.1] – 0.52[*I.BPMC] – 0.52[*F-plant noncoverage] – 0.83[*concrete bank]

Uncontracted best model ($r^2 = 0.75$)

E(ln(*y*)) = 3.18 – 0.39[I.BPMC] – 0.40[F-plant noncoverage] – 1.03[concrete bank] + 0.62[F.fthalide] + 0.60[area]

Small animal (with interaction)

Contracted best model ($r^2 = 0.77,\ n_{\mathrm{model}} = 27$)

E(ln(*y*)) = 3.87 – 0.61[Cont.var1.1] – 0.33[bullfrog] – 0.45[*F-plant noncoverage] – 1.31[*I.BPMC XXX concrete bank] – 0.72[F-plant noncoverage XXX concrete bank]

Uncontracted best model ($r^2 = 0.82$)

E(ln(*y*)) = 2.91 – 0.22[bullfrog] – 1.01[I.BPMC XXX concrete bank] – 0.80[F-plant noncoverage XXX concrete bank] + 0.48[H.pentoxazone] + 0.35[area]

Vertebrate

Contracted best model ($r^2 = 0.23,\ n_{\text{model}} = 17$):

E(ln(y)) = 1.66 -0.76[F.Probenazole]

Uncontracted best model ($r^2 = 0.43$):

E(ln(y)) = 1.70 -0.58[F.Probenazole] -0.58[Black_bass]

Invertebrate

Contracted best model ($r^2 = 0.72,\ n_{\text{model}} = 4$):

E(ln(*y*)) = 4.85 – 0.37[Cont.var1.1] – 0.39[Cont.var1.3] – 0.71[*I.BPMC] – 0.65[shallowness] – 0.67[*F-plant noncoverage] – 0.58[%concrete bank]

Uncontracted best model ($r^2 = 0.75$):

E(ln(*y*)) = 4.14 + 0.36[I.thiamethoxam] + 0.46[H.butachlor] – 0.65[I.BPMC] – 0.58[shallowness] – 0.60[F-plant noncoverage] – 0.64[concrete bank]

Invertebrate (with interaction)

Contracted best model ($r^2 = 0.74,\ n_{\text{model}} = 4$):

E(ln(*y*)) = 4.76 – 0.63[Cont.var1.1] – 0.30[bullfrog] – 0.59[shallowness] – 0.59[F-plant noncoverage] – 1.25[*I.BPMC XXX concrete bank] – 0.63[%F-plant noncoverage XXX concrete bank]

Uncontracted best model ($r^2 = 0.75$):

E(ln(*y*)) = 4.12 + 0.58[I.thiamethoxam] – 0.32[bullfrog] – 0.54[shallowness] – 0.58[F-plant noncoverage] – 1.19[I.BPMC XXX concrete bank] – 0.38[F-plant noncoverage XXX concrete bank]

Fish

Contracted best model ($r^2 = 0.23,\ n_{\text{model}} = 15$):

E(ln(*y*)) = 1.35 – 1.03[*F.probenazole]

Uncontracted best model ($r^2 = 0.68$):

E(ln(y)) = 1.37 – 0.87[F.probenazole] – 4.67[black bass] + 4.87[I.tebufenozide] + 4.40[F.fthalide] – 1.42[F.isoprothiolane]

Large insect

Contracted best model ($r^2 = 0.86,\ n_{\text{model}} = 7$):

E(ln(y)) = 4.61 – 1.11[bluegill] – 0.96[Cont.var1.3] – 0.68[crayfish] – 2.12[I.BPMC] – 1.73[F-plant noncoverage] – 0.83[concrete bank]

Uncontracted best model ($r^2 = 0.85$):

E(ln(y)) = 3.93 – 0.97[*bluegill] + 0.93[H.butachlor] – 0.70[crayfish] – 2.14[*I.BPMC] – 1.78[*F-plant noncoverage] – 1.02[concrete bank]

Large insect (with interaction)

Contracted best model ($r^2 = 0.88,\ n_{\text{model}} = 4$):

E(ln(y)) = 5.85 – 0.79[Cont.var1.1] – 1.73[Cont.var1.3] – 0.78[crayfish] – 1.53[%shallowness] – 1.76[*F-plant noncoverage] – 2.58[*bluegill XXX I.BPMC]

Uncontracted best model ($r^2 = 0.92$):

E(ln(y)) = 6.00 + 1.76[H.butachlor] – 0.83[crayfish] – 1.56[shallowness] – 2.18[F-plant noncoverage] – 2.24[bluegill XXX I.BPMC] – 1.97[E-plant noncoverage]

Small insect

Contracted best model ($r^2 = 0.58,\ n_{\text{model}} = 39$):

E(ln(y)) = 2.63 + 0.41[F.probenazole] – 0.43[F-plant noncoverage] – 0.74[concrete bank]

Uncontracted best model ($r^2 = 0.77$):

E(ln(y)) = 2.57 – 1.03[*concrete bank] – 0.95[TN] + 0.93[area] – 0.48[F.isoprothiolane]

S2.8 Impacts of statistically contributive explanatory variables

When the contracted best model had K explanatory variables, of which J variables had

statistically contributive effects, we calculated their impacts on the response variable (taxonomic richness of the focal animal category) as follows. We permuted the explanatory variables so that the statistically contributive variables come first, which allowed rewriting of Eq. (2) in the main text as

$$\ln(Y_i) \;=\; \alpha + \sum_{j=1}^{J} \beta_j x_{j,i} + \sum_{k=J+1}^{K} \beta_k x_{k,i} + r_i. \tag{S2.9}$$

We assumed a hypothetical 0th pond with all contributive variables having zero intensities and all non-contributive variables having the average intensities among the studied ponds (i.e., $x_{j,0} = 0$ for all $j = 1, \dots, J$, and $x_{k,0} = \bar{x}_k = \frac{1}{M}\sum_{i=1}^{M} x_{k,i}$ for all $k = J + 1, \dots, K$). We call this hypothetical pond the normal pond. From Eq. (S2.9), the expected taxonomic richness of the normal pond is given by

$$R = \exp\left(\alpha + \sum_{k=J+1}^{K} \beta_k \bar{x}_k\right), \tag{S2.10}$$

where $R = Y_0$ holds for the normal Poisson regression ($r_i = 0$). At the normal pond, if we increase the intensity of the jth explanatory variable, $x_{j,0}$, from its minimum value 0 to its average $\bar{x}_j$ among the studied ponds, then the expected taxonomic richness is given by $R_j^{\mathrm{mean}} = R\exp(\beta_j \bar{x}_j)$. The change rate of the taxonomic richness is calculated as $R_j^{\mathrm{mean}}/R = \exp(\beta_j \bar{x}_j)$. On this basis, we calculated the mean impact of the jth explanatory variable as the strength of the change rate,

$$I_j^{\mathrm{mean}} \;=\; \begin{cases} \dfrac{R_j^{\mathrm{mean}}}{R} = \exp(\beta_j \bar{x}_j) & \text{for } \beta_j > 0 \\ \dfrac{R}{R_j^{\mathrm{mean}}} = \exp(-\beta_j \bar{x}_j) & \text{for } \beta_j < 0. \end{cases} \tag{S2.11}$$

Note that I_j^{mean} for positive β_j indicates the strength of the increasing rate, whereas I_j^{mean} for negative β_j gives the strength of the diminishing rate.

Analogously, if we increase the intensity of the jth explanatory variable from its minimum value 0 to its maximum 1 at the normal pond, then the expected taxonomic richness is given by

$R_j^{\max} = R\exp(\beta_j)$. On this basis, we calculated the maximum impact of the jth explanatory variable as follows:

$$I_j^{\max} = \begin{cases} \dfrac{R_j^{\max}}{R} = \exp(\beta_j) & \text{for } \beta_j > 0 \\ \dfrac{R}{R_j^{\max}} = \exp(-\beta_j) & \text{for } \beta_j < 0. \end{cases} \quad \text{(S2.12)}$$

When all statistically contributive variables in the contracted best model had negative effects (i.e., $\beta_j < 0$ for all $j = 1, \ldots, J$), then by changing their intensities at the normal pond so that $x_{j,0} = x_{j,i}$ holds for all $j = 1, \ldots, J$, we calculated their combined negative impact at the ith pond as the strength of diminishing rate,

$$I_{\{1,\ldots,J\}}^{i} = \frac{R}{R\exp\left(\sum_{j=1}^{J}\beta_j x_{j,i}\right)} = \exp\left(-\sum_{j=1}^{J}\beta_j x_{j,i}\right). \quad \text{(S2.13)}$$

From this equation, we calculated the mean combined impact as a geometric mean among $I_{\{1,\ldots,J\}}^{i}$ for $j = 1, \ldots, J$, as

$$I_{\{1,\ldots,J\}}^{\text{mean}} = \left[\Pi_{i=1}^{M} I_{\{1,\ldots,J\}}^{i}\right]^{\frac{1}{M}} = \exp\left(-\sum_{j=1}^{J}\beta_j \bar{x}_j\right), \quad \text{(S2.14)}$$

which corresponds to the combined impact on the average pond. In addition, we calculated the maximum combined impact as the maximum among $I_{\{1,\ldots,J\}}^{i}$ for $j = 1, \ldots, J$ as

$$I_{\{1,\ldots,J\}}^{\max} = \max\left\{I_{\{1,\ldots,J\}}^{1}, \ldots, I_{\{1,\ldots,J\}}^{M}\right\}. \quad \text{(S2.15)}$$

When some of statistically contributive variables had positive effects, those variables were omitted. In this study, we also omitted statistically contributive explanatory variables that did not have statistically significant effects. (As for the combined impact of positive effects, its mean and maximum can be calculated with Eqs. (S2.13–S2.15) by removing the minus symbol on the right-hand sides of Eqs. (S2.13) and (S2.14) and omitting variables with negative effects instead, although such a calculation was not conducted in this study.)

S2.9 Discussion on our statistical method

In multivariate regression analysis, too many explanatory variables can lead to a

multicollinearity problem as well as extremely heavy calculation for model selection procedures. However, removing and/or aggregating some of those variables based on relevant previous studies may cause difficulty in detection of unknown relationships between the response and explanatory variables. To handle this difficulty, we developed a new statistical procedure for multivariate regression analysis by combining the contraction of explanatory variables (by using only correlations among them), best-subset model selection, stepwise model selection, and permutation tests. This procedure enabled us to detect previously unknown and significantly negative effects of two pesticides, probenazole (fungicide) and BPMC (insecticide), on taxonomic richness of the sampled animals and to evaluate the combined impacts of BPMC and other environmental stressors. In principle, our procedure is applicable to data with not only univariate response variables but also multivariate ones, as long as the models' AICs (or other suitable criteria) can be calculated.

In this study, the most statistically contributive stressors, those satisfying conditions (i–iii) defined in the "Statistical inference" section in the main text, were also statistically significant in the permutation test that explicitly repeats the model selection process. Thus, first finding statistically contributive explanatory variables and then examining their statistical significance may be an efficient strategy, because the permutation test that repeats model selection requires heavy calculation. Further examination and improvement of our procedure, and clarification of its relationships with other approaches for post-model-selection inference (Leeb et al. 2015; Taylor and Tibshirani 2015, 2018; Lee and Wu2018), may provide more efficient and robust tools for such inference.

References

Akaike H. Information theory and an extension of the maximum likelihood principle. In: Petrov BN, Caski F, editors. Proceedings of the 2nd International Symposium on Information Theory. Budapest: Akadimiai Kiado; 1973. pp. 267-281.

Broström G, Holmberg H. Generalized linear models with clustered data: Fixed and random effects models. Computational Statistics & Data Analysis 2011;55: 3123-3134.

Clarke KA. Nonparametric model discrimination in international relations. Journal of Conflict Resolution 2003;47:72–93.

Cox DR. Tests of separate families of hypotheses. Proceedings of the Fourth Berkeley Symposium on Mathematical Statistic and Probability 1961;1:105–123.

Kitagawa M, Sakamoto Y, Ishiguro M, Kitagawa G. Entropy statistics (in Japanese). Tokyo: Kyoritsu Press; 1983.

Lee SMS, Wu Y. A bootstrap recipe for post-model-selection inference under linear regression models. Biometrika 2018;105: 873-890. https://doi.org/10.1093/biomet/asy046.

Leeb H, Pötscher BM, Ewald K. On various confidence intervals post-model-selection. Statistical Science 2015;30: 216-227.

Murtaugh PA. In defense of P values. Ecology 2014;95:611–617. doi:10.1890/13-0590.1.

Taylor J, Tibshirani RJ. Statistical learning and selective inference. Proceedings of the National Academy of Sciences USA 2015;112(25): 7629-7634. doi: http://www.pnas.org/content/112/25.

Taylor J, Tibshirani R. Post-selection inference for l_1 penalized likelihood models. The Canadian Journal of Statistics 2018;46: 41-61.

Vuong QH. Likelihood ratio tests for model selection and non-nested hypotheses. Econometrica 1989;57(2):307–333.

Werft W, Benner A. glmperm: a permutation of regressor residuals test for inference in generalized linear models. R Journal 2010;2(1): 39-43.
