# Supplementary figures and images for: Combined impact of pesticides and other environmental stressors on animal diversity in irrigation ponds
Source: PLoS One. 2020 Jul 2;15(7):e0229052. doi: 10.1371/journal.pone.0229052 (PMC7332035; doi:10.1371/journal.pone.0229052)

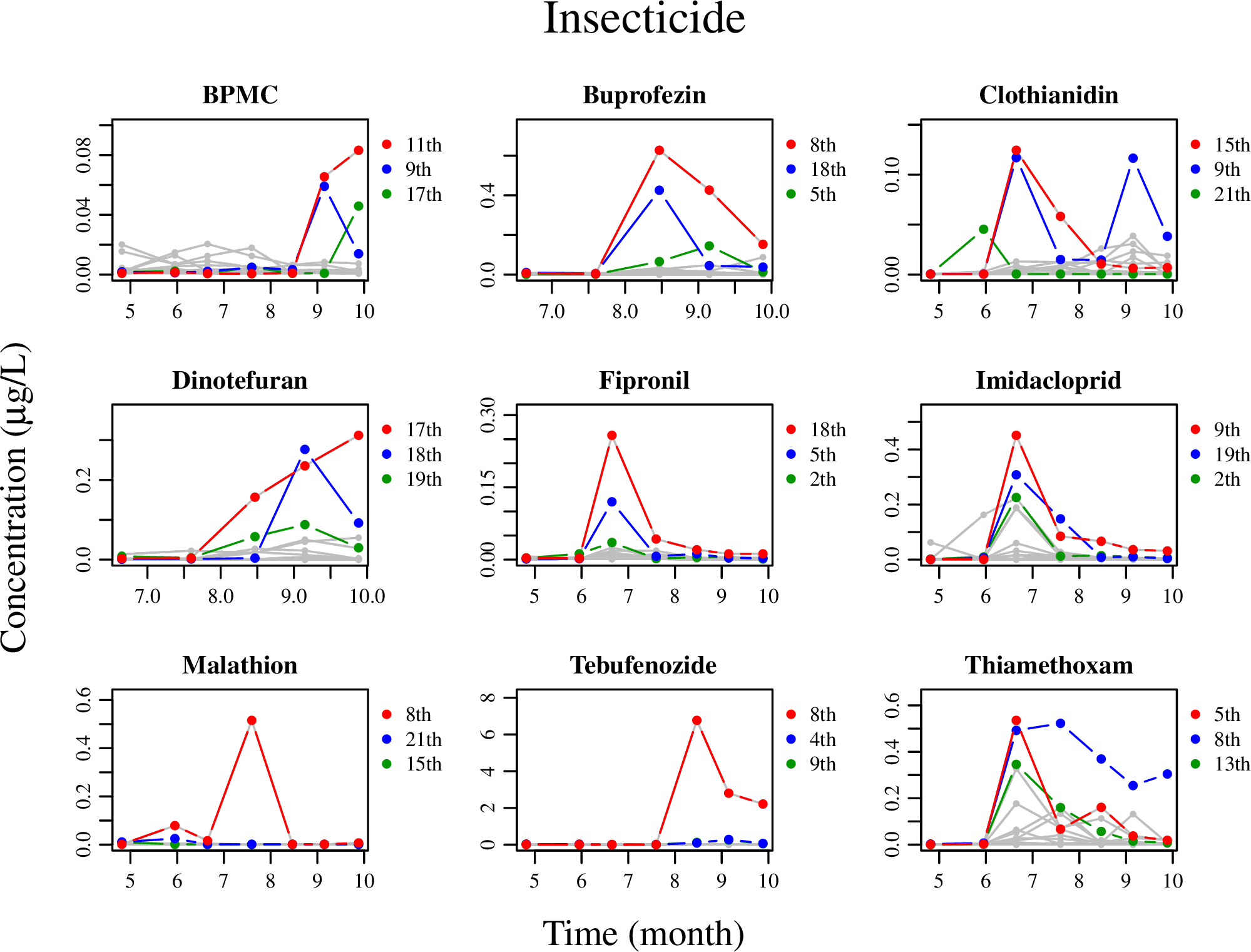

Supplement: S1 Fig — In each panel, red, blue, and green indicate the top 3 ponds with the highest detected concentrations among the 21 ponds. The others are colored gray. Each point connecting line segments indicates one of the seven samplings during the study period. (TIF) [file pone.0229052.s004.tif]

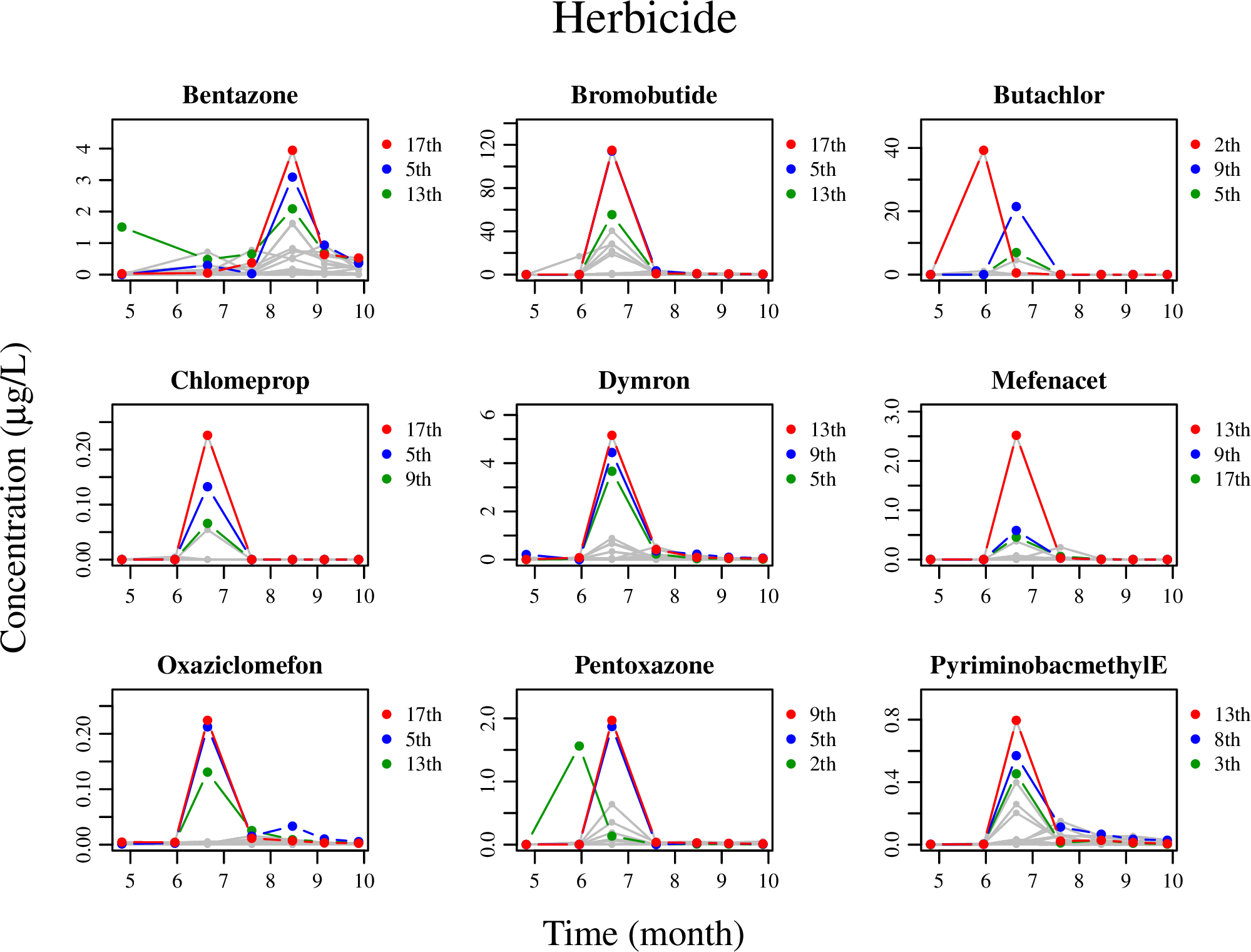

Supplement: S2 Fig — The plotting was done as in S1 Fig. (TIF) [file pone.0229052.s005.tif]

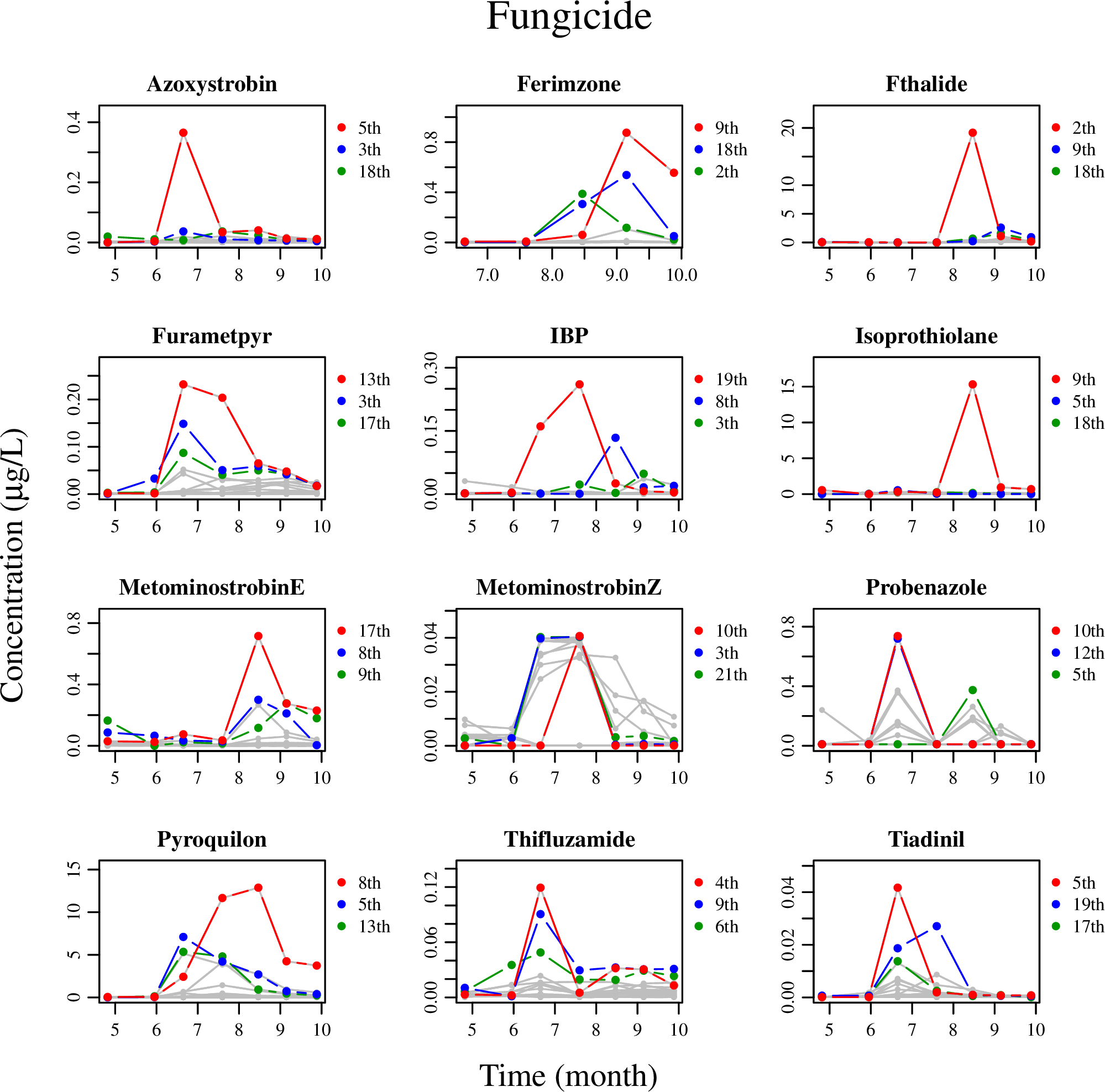

Supplement: S3 Fig — The plotting was done as in S1 Fig. Among the 13 fungicides measured, TPN is not shown because it was not detected in any pond. (TIF) [file pone.0229052.s006.tif]

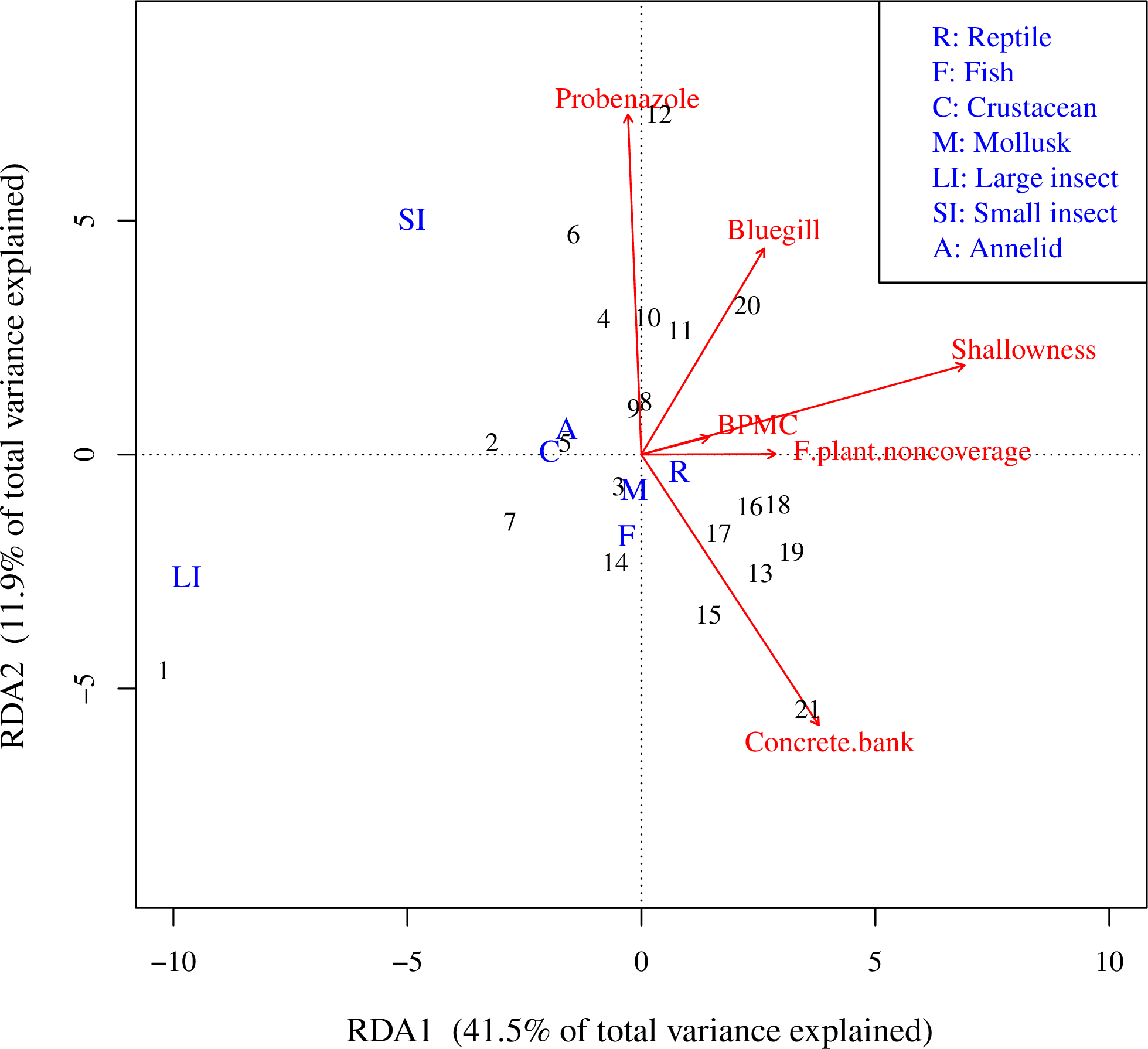

Supplement: S4 Fig — Plotted numbers indicate pond IDs. 55.6% of total variance was explained by all RDA axis. The calculation was conducted by R (version 3.4.4) and its package “vegan” (version 2.5–3). (TIF) [file pone.0229052.s007.tif]
